# A Suit for Fees by a Clairvoyant

**Published:** 1846-02

**Authors:** 


### A SUIT FOR FEES BY A CLAIRVOYANT.

The Boston Medical and Surgical Journal copies from a New York paper an amusing account of an action brought by a clairvoyant for medical services. Livingston and Davis the plaintiffs, practising upon the plan of medical clairvoyance, had attended the family of David Jackson, examining and prescribing for his wife and daughter. The bill claimed was $50. Davis, according to the witnesses, was put to sleep, by his associate, when he proceeded to examine the patient, and describe the remedies to be administered; these being written down were invariably given, Livingston himself never examining the patient, and the clairvoyant doing so only in his mesmeric state, and with a handkerchief over his eyes.

The court decided in favor of the defendant, principally, it is stated, "on the ground of the deception and fraud of the system."

